# High-Throughput Sequencing and Co-Expression Network Analysis of lncRNAs and mRNAs in Early Brain Injury Following Experimental Subarachnoid Haemorrhage

**DOI:** 10.1038/srep46577

**Published:** 2017-04-18

**Authors:** Jianhua Peng, Yue Wu, Xiaocui Tian, Jinwei Pang, Li Kuai, Fang Cao, Xinghu Qin, Jianjun Zhong, Xinshen Li, Yong Li, Xiaochuan Sun, Ligang Chen, Yong Jiang

**Affiliations:** 1 Department of Neurosurgery, the Affiliated Hospital of Southwest Medical University, Luzhou, China; 2 Department of Neurosurgery, the First Affiliated Hospital of Chongqing Medical University, Chongqing, China; 3 Chongqing Key Laboratory of Biochemistry and Molecular Pharmacology, College of Pharmacy, Chongqing, China; 4 Department of Ophthalmology, the Affiliated Hospital of Southwest Medical University, Luzhou, Sichuan, China; 5 Department of Neurovascular Disease, the Affiliated Hospital of Zunyi Medical College, Zunyi, China; 6 Department of Neurosurgery, People’s Hospital of Deyang City, Deyang, China

## Abstract

Subarachnoid haemorrhage (SAH) is a fatal neurovascular disease following cerebral aneurysm rupture with high morbidity and mortality rates. Long non-coding RNAs (lncRNAs) are a type of mammalian genome transcript, are abundantly expressed in the brain and are involved in many nervous system diseases. However, little is currently known regarding the influence of lncRNAs in early brain injury (EBI) after SAH. This study analysed the expression profiles of lncRNAs and mRNAs in SAH brain tissues of mice using high-throughput sequencing. The results showed a remarkable difference in lncRNA and mRNA transcripts between SAH and control brains. Approximately 617 lncRNA transcripts and 441 mRNA transcripts were aberrantly expressed at 24 hours after SAH. Gene ontology (GO) enrichment and Kyoto Encyclopedia of Genes and Genomes (KEGG) analysis indicated that the differentially expressed mRNAs were mostly involved in inflammation. Based on the lncRNA/mRNA co-expression network, knockdown of fantom3_F730004F19 reduced the mRNA and protein levels of CD14 and toll-like receptor 4 (TLR4) and attenuated inflammation in BV-2 microglia cells. These results indicate that lncRNA fantom3_F730004F19 may be associated with microglia induced inflammation via the TLR signaling pathway in EBI following SAH. LncRNA represent a potential therapeutic target for the prognosis, diagnosis, and treatment of SAH.

Subarachnoid haemorrhage (SAH) is a devastating cerebrovascular disease. Although SAH accounts for only 5% of all strokes, this type of haemorrhage affects a significant percentage of the population worldwide with high morbidity and mortality^1^,^2^. Early brain injury (EBI) refers to the acute pathophysiological event that occurs within the first 72 h of SAH. Differ from the fact that the reversal of cerebral vasospasm (CVS) does not appear to improve outcome, EBI has been demonstrated to be the main cause of death or disability in SAH. Therefore, to a large extent, EBI determines the prognosis of SAH patients^3^. Despite the vast efforts made in EBI research following SAH, the mechanisms of the pathological process in the acute phase of SAH require further investigation^4^.

Long non-coding RNAs (lncRNAs) are a type of RNA transcript lacking protein-coding potential (>200 nucleotides). For some lncRNAs, a high degree of tissue specificity has been identified. Remarkably, lncRNAs are expressed abundantly in the nervous system, and approximately 40% lncRNAs are detected specifically in the brain^5^,^6^,^7^. Previous studies have demonstrated that lncRNAs play important roles in regulating the pathological processes of neurological and psychiatric diseases^8^,^9^,^10^. To date, however, lncRNA expression signatures and the co-expression network of lncRNAs and mRNAs in EBI after SAH remain poorly understood. Furthermore, the function of lncRNAs in EBI following SAH also requires further study.

It is imperative that appropriate animal models of SAH are used for research. The current SAH models mainly utilize endovascular perforation and blood injection techniques. However, the endovascular perforation model better mimics EBI, while the blood injection model better mimics vasospasm^11^,^12^. Therefore, to reveal the potential role of lncRNAs in EBI after SAH, we performed lncRNA expression profiling and co-expression network analysis of lncRNAs and mRNAs after SAH in an endovascular perforation mouse model using next-generation high-throughput sequencing. Based on the bioinformatics and co-expression analysis, we found that fantom3_F730004F19, a differentially expressed lncRNA, may be correlated with CD14/toll-like receptor 4 (TLR4)-related neuroinflammation. Silencing of lncRNA fantom3_F730004F19 by lentiviral vectors was used to confirm this hypothesis.

## Results

### Induced SAH model and RNA-seq quality control

**Figure 1:**
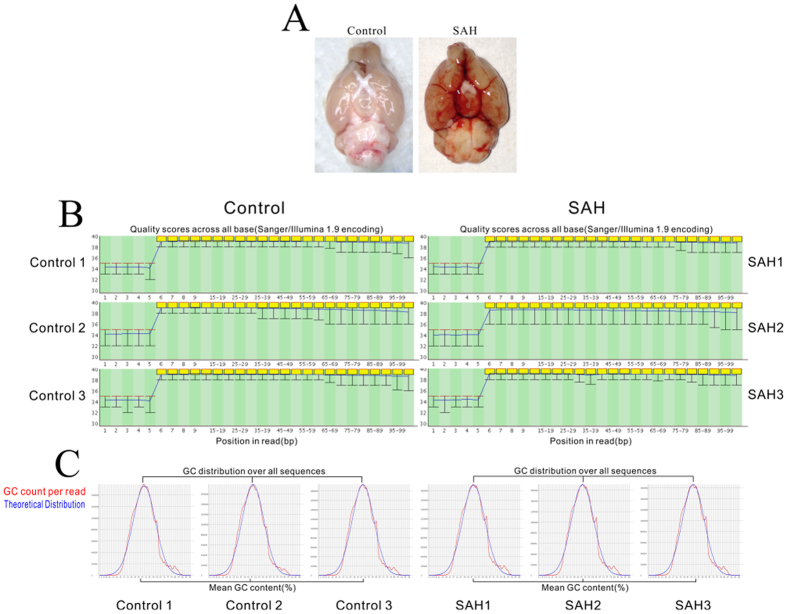
Induced SAH model and quality assessment of the gene expression levels in the control group and SAH group. (**A**) Images of mouse brains between in the control and SAH groups. There were no blood clots in the sham-operated mouse brains, while a thick blood clot was found around the arterial circle of Willis after the SAH operation. (**B**) As shown in the box plot, almost all of the quality scores (−10*log10 (*P-*value)) were approximately 40 in all samples. (**C**) The Gaussian distribution curve indicates the average GC content in all reads of one sample, the red line represents actual content, and the blue line represents the theoretical content. The real GC content of all samples showed a Gaussian distribution, which nearly agreed with the theoretical content. SAH, subarachnoid haemorrhage; GC, guanine-cytosine.

**Figure 2:**
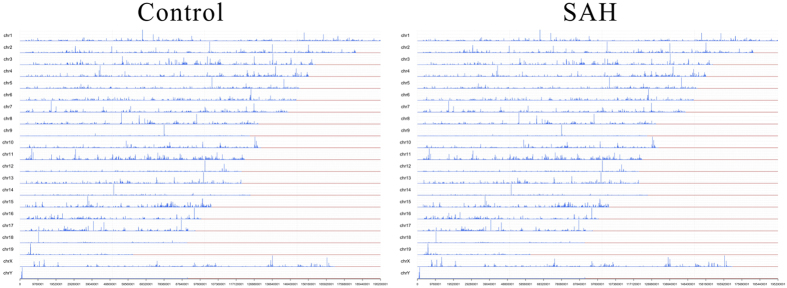
Representative mapping of sequence reads in the Control and SAH groups. Overall coverage of the detected reads was mapped to mouse chromosomes.

Twenty-four hours after surgery, both the control and SAH animals were sacrificed, and brain samples were removed rapidly. Blood clots were evident in the ventral subarachnoid space of SAH mice but not in that of the control animals (Fig. 1A). The box plot (Fig. 1B) and Gaussian distribution of GC sequence content (Fig. 1C) provided an overview of all normalized genes in all the samples, which suggested that the distributions of the intensities from all the samples were almost identical. All of the reads were optimized, and the cleaned data were mapped to mouse chromosomes (Fig. 2) and centred on several different regions as follows: exons (78.73%), introns (16.27%), intergenic regions (4.73%), and splicing regions (0.27%) in the sample representing the control group, compared to exons (82.94%), introns (12.11%), intergenic regions (4.66%), and splicing regions (0.29%) in the sample representing the SAH group (Table 1).

### Differentially expressed lncRNAs and mRNAs

**Figure 3:**
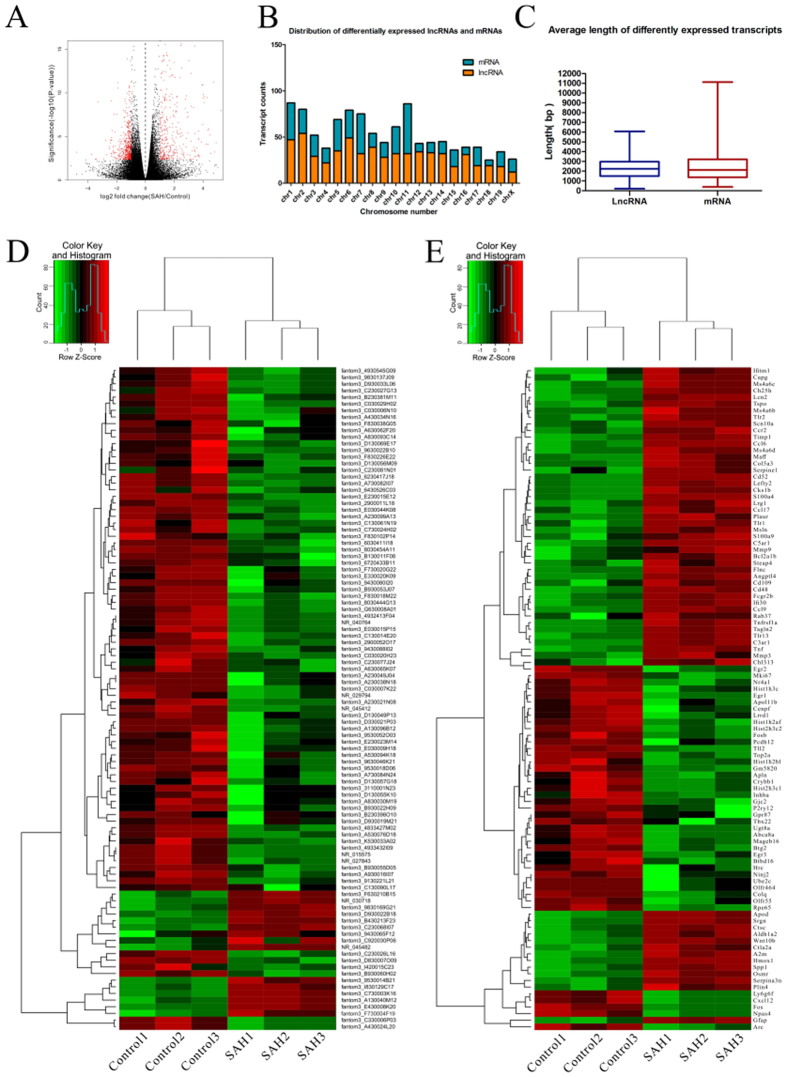
Differentially expressed lncRNAs and mRNAs between mouse brains at 24 h after SAH and in control groups. (**A**) The volcano plot illustrates the differentially expressed genes (including lncRNAs and mRNAs) between the control and SAH groups, with red dots representing differentially expressed genes (log2fold change > 1; *q* < 0.05). (**B**, **C**) Chromosome distribution (**B**) and average length (**C**) of the differentially expressed lncRNAs and mRNAs. (**D**,**E**) The hierarchical cluster analysis generated heat maps of the top 100 significant differential expressed lncRNAs (**D**) and mRNAs (**E**) in the mouse brains at 24 h following SAH or in control groups (log2fold change >1.5; *q* < 0.05). Colour represents the log10 (RPKM + 1) value, with red indicating upregulated genes and green indicating downregulated genes. The legend represents the standard normal distribution of log10 (RPKM + 1) values, and the blue line in the legend indicates the gene amounts in this colour area. SAH, subarachnoid haemorrhage; lncRNAs, long non-coding RNAs; mRNAs, messenger RNAs; RPKM, expected number of Reads Per Kilobase of transcript sequence per Millions base pairs Sequenced.

All RNA samples were subjected to high-throughput sequencing analysis of lncRNA and mRNA expression. All the sequencing data were filtered using the volcano plot to illustrate the differentially expressed coding genes and non-coding genes (log2fold change >1; *q* < 0.05) (Fig. 3A). All of the significant differentially expressed genes were distributed among almost all of the chromosomes (Fig. 3B). The average lncRNA transcript was 2,289 bp, and the average mRNA transcript was 2,497 bp (Fig. 3C; Supplementary Table S2; Supplementary Table S3). Among all significant differentially expressed genes in SAH and control samples (log2fold change > 1; *q* < 0.05), there were 103 upregulated and 514 downregulated lncRNA transcripts (Fig. 3D) and 387 upregulated and 54 downregulated mRNA transcripts (Fig. 3E), respectively. In addition, fantom3_F730004F19, one of the upregulated lncRNAs in SAH brains compared with levels in the controls (fold change: 15.82, log2fold change: 3.98; *P* < 0.05), showed the closest correlation with a significant differentially expressed mRNA (CD14, fold change: 2.51, log2fold change: 1.33; *P* < 0.05) in the co-expression network of lncRNA transcripts and mRNA transcripts. Moreover, many transcripts did not align with any known mRNA or lncRNA, and their coding potential was analysed. Those transcripts longer than 200 bp but without coding potential were identified as lncRNAs. A total of 150 newly identified lncRNAs were differentially expressed between the control group and SAH group. The top differentially expressed transcripts are shown in Table 2.

### GO and KEGG analysis

**Figure 4:**
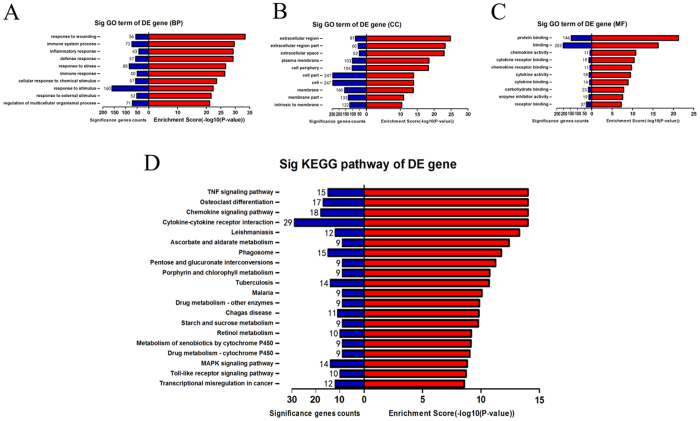
GO enrichment and KEGG pathway analysis of differentially expressed genes in SAH brains. (**A**) BP, (**B**) CC and (**C**) MF presented the top 10 significance terms of GO enrichment analysis (*P* < 0.05 and FDR < 0.05). (**D**) The top 20 KEGG pathways of significantly differentially expressed genes between the control and SAH groups (*P* < 0.05 and FDR < 0.05). GO, Gene Ontology; KEGG, Kyoto Encyclopedia of Genes and Genomes; BP, biological processes; CC, cellular components; MF, molecular functions; FDR, false discovery rate; DE, differentially expressed.

The function of lncRNAs is thought to be reflected in their associated protein-coding genes^13^. To clarify the potential function of these differentially expressed mRNA-related lncRNAs, gene ontology (GO) enrichment analysis of differentially expressed mRNAs was used. Three domains (biological processes, cellular components and molecular functions) of GO enrichment analysis were investigated. The significance of GO analysis in each domain was denoted by the false discovery rate (FDR) (FDR < 0.05 is recommended) (Fig. 4A–C). The response to wounding, immune system process, and inflammatory response were the most enriched terms in biological processes (BP) (Fig. 4A). Extracellular region, extracellular region part and extracellular space were the most enriched terms in cellular components (CC) (Fig. 4B). Protein binding, binding and chemokine activity were the most enriched terms in molecular functions (MF) (Fig. 4C). Kyoto Encyclopedia of Genes and Genomes (KEGG) pathway analysis indicated that the most enriched pathways involving significant differential expressed mRNAs were the tumour necrosis factor (TNF) signaling pathway, osteoclast differentiation and chemokine signaling pathway (Fig. 4D).

### LncRNA/mRNA transcripts co-expression networks

**Figure 5:**
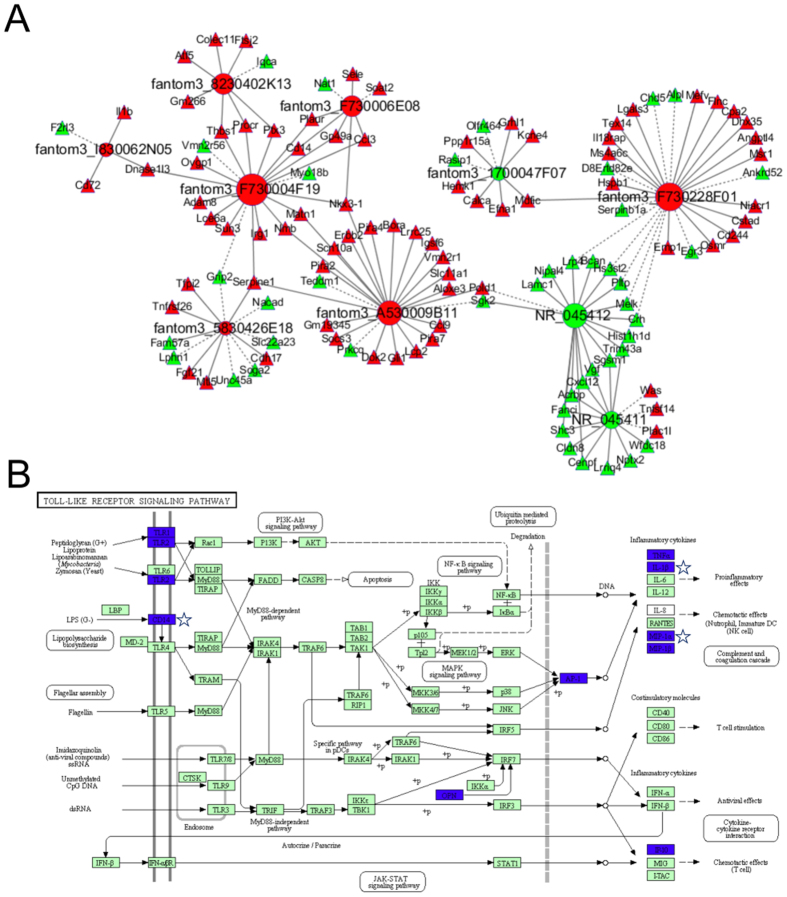
LncRNA/mRNA transcripts co-expression network after SAH. (**A**) The network represents the co-expression correlations between the significantly differentially expressed mRNA and lncRNA transcripts (|COR| > 0.95, *P* < 0.05). Circles indicate lncRNA transcripts and triangles indicate mRNA transcripts. Solid lines indicate positive correlations, and dashed lines indicate negative correlations. Red represents upregulated, and green represents downregulated. (**B**) KEGG analysis suggested that lncRNA-coexpressed mRNAs were mainly targeted to the Toll-like receptor signaling pathway (http://www.genome.jp/kegg)^54^,^55^. Blue represents significant genes in this pathway of SAH, and pentagrams represent significant lncRNA-coexpressed genes.

To reveal the key lncRNAs and their potential functions in SAH, we constructed a lncRNA/mRNA co-expression network, and investigated the potential interactions between the lncRNA transcripts and mRNA transcripts. More than 1,800 network nodes were composed. Ten network nodes with high correlation (COR)-values were selected, and the co-expression network was established using Cytoscape software (Fig. 5A). Most of the lncRNA-coexpressed mRNA transcripts were involved in the inflammatory response (biological process), extracellular region (cellular component) and protein binding (molecular function) according to GO enrichment. Moreover, we found that CD14, a ligand for toll-like receptor 4 (TLR4), which is involved in regulation of the TLR signaling pathway, showed the closest correlation with one lncRNA (fantom3_F730004F19) among all significant differentially expressed mRNA transcripts with the highest COR-value (COR: 0.999, *P* < 0.05) (Fig. 5B; Supplementary Table S4).

### qRT-PCR validation

**Figure 6:**
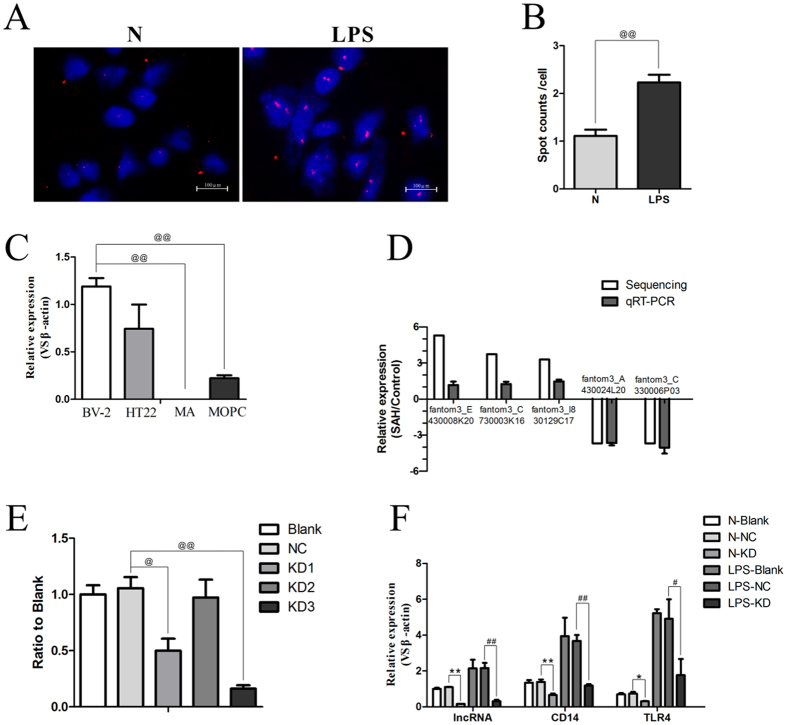
Gene expression profile. (**A**,**B**) Subcellular localization pattern of fantom3_F730004F19 by RNA FISH staining. A significantly higher expression of fantom3_F730004F19 in treated BV-2 microglia cells was estimated, and fantom3_F730004F19 was predominantly expressed in the nucleus. (**C**) Cellular localization pattern of fantom3_F730004F19 by qRT-PCR. Although there was no statistical difference between BV2 and HT22 cells, the level of fantom3_F730004F19 present a higher in BV2 cell line, lesser extent in HT22 cells and MOPC cells, and not detected in MA cells. (**D**) Comparison between sequencing and qRT-PCR analysis of 5 randomly selected lncRNAs. Positive values refer to upregulation, and negative values refer to downregulation. *β*-actin was used to normalize the expression of samples. Red representes RNA-seq data, and blue representes qRT-PCR. The bars represent standard error of the mean (SEM). The qRT-PCR results were closely correlated with the sequencing data (*P* < 0.05). (**E**) The silencing efficiency of the lentivirused on fantom3_F730004F19 in BV-2 microglia cells. Lentivirus-50305 (KD1) and lentivirus-50307 (KD3) significantly inhibited fantom3_F730004F19 expression (*P* < 0.05 versus Blank group). (**F**) Inhibition of fantom3_F730004F19 reduced the expression of CD14 and TLR4 in BV-2 microglia cells following LPS treatment. The expression of fantom3_F730004F19, CD14 and TLR4 were overexpressed in BV-2 cells after LPS treatment. Knockdown of fantom3_F730004F19 abated the increase of CD14 and TLR4 in LPS-treated BV-2 cells. ^@^*P* < 0.05 and ^@@^*P* < 0.01; *P < 0.05 and **P < 0.01 versus the NC normal group; ^#^*P* < 0.05 and ^##^*P* < 0.01 versus the NC LPS-treated group. BV-2: mouse microglial cell line; HT22: mouse neuronal cell line; MA: mouse astrocyte cell line; MOPC: mouse oligodendrocyte precursor cell line; N: normal condition group; LPS: lipopolysaccharide treated; Blank: non-transfected cells; NC: negative control lentivirus transfected cells; KD: the lncRNA fantom3_F730004F19 lentivirus transfected cells.

To validate the RNA-seq data, qRT-PCR was performed using 5 randomly selected lncRNAs (3 upregulated and 2 downregulated). qRT-PCR analysis revealed that the expression levels of fantom3_E430008K20, fantom3_C730003K16 and fantom3_I830129C17 were upregulated, while those of fantom3_A430024L20 and fantom3_C330006P03 were downregulated in SAH brains. The qRT-PCR results confirmed the accuracy of RNA-seq (Fig. 6D).

### Cellular and subcellular localization pattern of fantom3_F730004F19

Mouse microglial cell line BV-2 cells, neuronal cell line HT22 cells, astrocyte cell line MA cells and oligodendrocyte precursor cell line MOPC cells were used to identify the distribution of fantom3_F730004F19 in cells of the central nervous system (CNS). Although there was no statistical difference between BV2 and HT22 cells, qRT-PCR indicated BV2 cell line presented a higher level of fantom3_F730004F19 (Fig. 6C). After treatment with lipopolysaccharide (LPS) in BV-2 microglia cells, a significantly higher expression level of fantom3_F730004F19 was identified with RNA FISH staining. Additionally, fantom3_F730004F19 was predominantly expressed in the microglial nuclei (Fig. 6A,B).

### Knockdown of fantom3_F730004F19 reduced the expression of inflammation-related genes

Three different lentiviruses were transfected into BV-2 cells to inhibit fantom3_F730004F19. The expression of fantom3_F730004F19 was significantly reduced after transfection with lentiviral vectors. Compared with the levels in the negative control (nonsense lentivirus, NC), lentivirus-50305 (KD1) and lentivirus-50307 (KD3) reduced the fantom3_F730004F19 levels by > 50% (*P* < 0.05) (Fig. 6E). Lentivirus-50307 (KD3) presented a specific lentiviral-mediated knockdown of fantom3_F730004F19 (Supplementary Figure S3). We next employed lentivirus-50307 (KD3) to inhibit fantom3_F730004F19 expression for subsequent functional analysis. After treatment of BV-2 microglia cells with LPS, the levels of CD14 and TLR4 mRNA were sharply increased, and fantom3_F730004F19 expression was dramatically upregulated (*P* < 0.05). Lentivirus (KD3) treatment significantly suppressed the expression of fantom3_F730004F19 in both the normal group and the LPS group, and the elevation in CD14 and TLR4 mRNA was also inhibited (*P* < 0.05) (Fig. 6F). These findings suggested that fantom3_F730004F19 regulated the levels of CD14 and TLR4 mRNA.

### Knockdown of fantom3_F730004F19 attenuated the inflammatory response

**Figure 7:**
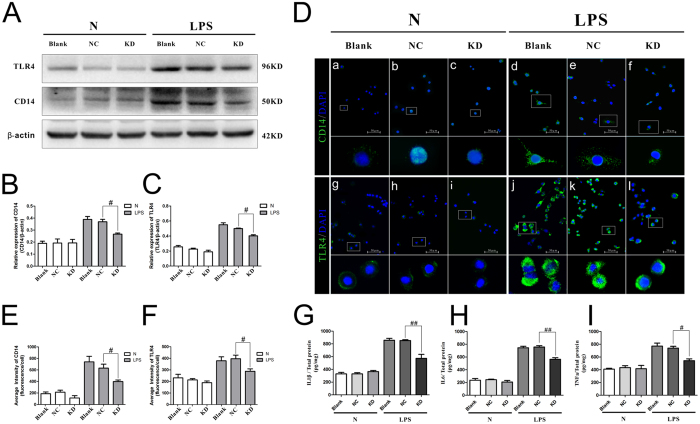
Silencing fantom3_F730004F19 attenuated the inflammatory response. (**A**–**F**) Inhibition of fantom3_F730004F19 reduced the expression of CD14 and TLR4 in BV-2 cells following LPS treatment. Protein expression was shown to be completely depleted by both western blot analysis (**A**–**C**) and immunofluorescence staining (**D**–**F**) (full-length blots/gels are presented in Supplementary Figure S5). Although the inhibitory effect was not obvious in the normal condition, silencing fantom3_F730004F19 significantly reduced the expression of CD14 and TLR4 protein levels after LPS treatment (^#^*P* < 0.05 and ^##^*P* < 0.01 versus the NC LPS-treated group). (**G**–**I**) Inhibition of fantom3_F730004F19 attenuated inflammatory cytokines release. Silence fantom3_F730004F19 significantly reduced the release of IL-1β (**G**), IL-6 (**H**) and TNF-α (**I**) after LPS treatment (^#^*P* < 0.05 and ^##^*P* < 0.01 versus the NC LPS-treated group). N: normal condition group; LPS: lipopolysaccharide treated; Blank: non-transfected cells; NC: negative control lentivirus transfected cells; KD: the lncRNA fantom3_F730004F19 lentivirus transfected cells.

Compared with the levels in the Blank and NC groups, the expression levels of CD14 and TLR4 proteins were dramatically suppressed after lentivirus treatment in LPS-treated BV-2 microglia cells (*P* < 0.05), but, this effect was not observed in normal conditions (Fig. 7A–F). We further quantified the protein levels of TNF-α, IL-1β and IL-6 in the BV-2 supernatants. After lentivirus treatment, the protein levels of IL-1β (Fig. 7G), IL-6 (Fig. 7H) and TNF-α (Fig. 7I) were significantly suppressed in the LPS group (*P* < 0.05). These findings indicated that knockdown of fantom3_F730004F19 attenuated microglia-related inflammation.

## Discussion

In summary, significantly differentially expressed lncRNA and mRNA transcripts were examined in the present study. We identified 617 lncRNAs and 441 mRNAs that were aberrantly expressed at 24 h after SAH. KEGG and GO analysis revealed that these differentially expressed mRNA genes involved in many pathophysiological processes, including the inflammatory responses. Meanwhile, the vast majority of lncRNA-coexpressed mRNAs were also associated with inflammation. Specifically, we found that among all lncRNAs, lncRNA fantom3_F730004F19 has the closest relationship with differentially expressed mRNA transcripts, which was correlated with CD14 at the highest COR value (COR: 0.999). Silencing of fantom3_F730004F19 resulted in reduced expression of CD14 and TLR4 at both the mRNA and protein levels. Moreover, knockdown of fantom3_F730004F19 attenuated inflammation in BV-2 microglia cells. These results provide initial experimental evidence that lncRNAs may have some specific effects on the pathological processes of EBI in SAH by regulating inflammation.

The neuroinflammatory response in EBI after SAH has been identified in both clinical and experimental studies^14^,^15^,^16^. Accordingly, anti-inflammatory intervention is a novel promising area of research for SAH treatment^11^. Inflammatory cytokines, such as TNF-α and IL1β, are strongly associated with brain injury after SAH and have been demonstrated to exert inflammatory and excitotoxic effects^17^, initiate neuronal apoptosis^18^, activate matrix metalloprote 9 (MMP-9) and cause blood brain barrier (BBB) disruption^19^,^20^. We systematically analysed the functions of differentially expressed mRNAs by GO annotation and pathway analysis. Our experimental findings revealed inflammatory responses in the mouse brain at 24 h after SAH. Significantly differentially expressed genes related to cytokine pathways were observed in our experiment, including Il1b and Tnf (gene names). The genes of chemokine family, including Ccl2 (also known as MCP1), Ccl3 (also known as MIP-1α), Ccl4 (also known as MIP-1β), which have been shown to be involved in inflammatory responses^21^,^22^, were also differentially expressed at 24 h after SAH. These results are consistent with the common view that neuroinflammation contributes to EBI after SAH.

Based on recent advances in genome sequencing techniques, studies targeting the roles of lncRNAs in inflammation and the immune response has been conducted^23^,^24^. In particular, aberrant lncRNA expression has been shown to be involved in neurological disorders, such as neurodegenerative diseases^25^, schizophrenia^26^, autism^27^, intellectual disability and developmental delay^28^,^29^. Zheng *et al*. elucidated the expression signatures of lncRNAs in a blood injection model using microarray assays^30^. However, the discovery rate of high-throughput sequencing is higher than that of microarray technology. To better mimic EBI after SAH^11^,^12^, we performed the endovascular perforation model. In contrast to the observations of Zheng *et al*.,which were made using microarray analysis with the blood injection model in rats, our sequencing data indicated that numerous of lncRNAs, such as lncRNA fantom3_F730004F19, were differentially expressed after SAH, which has not been previously reported. Based on data from our GO analysis, we constructed a co-expression network to further analyse the relationship between lncRNAs and deregulated mRNAs.

In particular, to reveal the function of lncRNAs, we performed the co-expression network according to the linear relationship and calculated the correlation coefficient as previously reported^31^. The co-expression network indicated that lncRNA fantom3_F730004F19 was highly correlated with CD14. Knockdown of lncRNA fantom3_F730004F19 reduced CD14 and TLR4 gene and protein levels and attenuated inflammation in BV-2 microglia cells subjected to LPS treatment. Furthermore, RNA FISH staining suggested that fantom3_F730004F19 was predominantly expressed in the microglia nucleus, which may be consistent with a role for these lncRNAs in chromatin regulation^32^. CD14 is a ligand of TLR4, which is regarded as a key organizer of microglial responses to CNS infection and injury^33^. Expressed at the cell surface with TLR4, LPS binds to the CD14 receptor^34^, activates TLR4 and initiates TLR4 signalings via the MyD88 and TRIF signaling pathways^35^,^36^. The damaging role of TLR4 signaling in experimental SAH has been investigated^37^, and lncRNAs are known to play pivotal roles in the modulation of the inflammatory response via TLRs signaling^38^. We further identify potential mRNA targets of fantom3_F730004F19. The results indicated that fantom3_F730004F19 has a cis-acting regulation target: Gp49a/Lilrb4. Although the role of Gp49a/Lilrb4 in TLR signaling pathway remains poorly understood, Gp49a/Lilrb4 suggests a suppressive/tolerizing influence of CD11c -positive microglia cells, and involves microglia activation and inflammatory/immune response^39^,^40^. Future works at the role of Gp49a/Lilrb4 in TLR signaling pathway and EBI are worth to investigate.

Our previous study indicated that a microglia-related inflammatory response leads to secondary injury in traumatic brain injury (TBI) and SAH^41^,^42^. Microglia are the resident immune cells in the CNS, and the effects of microglial activation in CNS disorders are under intense scrutiny. Indeed, activated microglia play dual roles^43^. In some situations, microglial activation is thought to benefit the injured brain by removing cellular debris and restoring tissue integrity^44^. Alternatively, microglia can be activated by blood content and polarized to the M1 phenotype via the TLR4 signaling pathway and exert inflammatory damage^45^,^46^. Although the depletion of microglia alleviates brain injury involving the TLR4 pathway after SAH^47^, the molecular mechanisms for TLR4-related microglial activation in the pathological processes of EBI remain poorly understood. Based on our observation, we speculate that changes in lncRNA fantom3_F730004F19 levels may affect the microglia-related inflammatory responses after SAH via modulation of the TLR signaling pathway, which may consequently influence the pathological processes of EBI.

There are several limitations of the current study. First, the pathophysiological process after SAH is dynamic^11^, and we only examined the expression of mRNAs and lncRNAs at 24 h after SAH. Future investigations at additional time-points are required to confirm the expression and potential functions of lncRNAs after SAH. Second, knockdown of fantom3_F730004F19 attenuated inflammation in BV-2 microglia cells. The specific mechanisms of fantom3_F730004F19 on inflammation require further exploration. Third, our sequencing data indicated that all significantly differentially expressed lncRNAs were evenly distributed in nearly all chromosomes, suggests that the lncRNA-mediated network plays a wide role in EBI pathogenesis after SAH. Nevertheless, additional clinical and experimental investigation aimed at the detailed functions of lncRNAs in SAH are required.

In conclusion, our study is the first to demonstrate associations between lncRNAs and mRNAs in an experimental SAH mouse model. LncRNAs were significantly differentially expressed in the mouse brain in the SAH model compared to control animals, and knockdown of fantom3_F730004F19 attenuated inflammation in BV-2 microglia cells. These results suggest that fantom3_F730004F19 may be involved in microglia-related inflammation in EBI after SAH. LncRNAs represent a promising potential therapeutic target for the prognosis, diagnosis, and treatment of SAH.

## Materials and Methods

### Ethics statement

The animal research protocol was approved by the Experimental Ethics Committee of Chongqing Medical University with the permit number: SYXK (Yu) 2012-001. All mice experimental procedures were performed in accordance with the Regulations for the Administration of Affairs Concerning Experimental Animals approved by the State Council of the People’s Republic of China, and every effort was made to minimize suffering.

### Animals

Adult (8–12 weeks) male C57BL/6 J mice were obtained from the Laboratory Animal Center of Chongqing Medical University. Mice were fed standard laboratory chow and supplied drinking water ad libitum.

### Induction of SAH

SAH induction was performed by endovascular perforation as previously described with slight modifications^48^. Mice were anesthetized with pentobarbital sodium (50 mg/kg) by intraperitoneal injection. After the mice were placed in a supine position, a midline incision was made on the neck, and the right common carotid artery (CCA) was exposed. Then, the CCA was traced cranially until the bifurcation, and the external carotid artery (ECA), and internal carotid artery (ICA) were exposed. A 5–0 prolene filament (Ethicon, Somerville, NJ, USA) was advanced via the ECA into the ICA. Then, the filament was gently pushed forward (≈12 mm) until some resistance was felt. The filament was advanced 1–2 mm further to perforate the anterior cerebral artery (ACA). The Cushing reflex indicated the successful induction of SAH. Subsequently, the filament was immediately withdrawn. Control group animals received the same procedure except for the perforation. Body temperature was maintained at 37 ± 0.5 °C throughout the procedure using a heating pad.

### RNA extraction and quality control

Twenty-four hours after SAH, mice (n = 18) were anesthetized and perfused with 100 mL cold PBS via the ascending aorta, followed by sacrifice. The clean cortex was quickly cut into small pieces, rinsed with PBS and dissolved with TRIzol (Invitrogen Life Technologies, Carlsbad, CA, USA). The lysates of three mice in each groups were pooled into one sample as previously described^49^. The concentration and quality of RNA was assessed by Nano Drop ND-1000 spectrophotometry (Nano Drop Technologies, Inc., Wilmington, DE, USA) and denatured for agarose gel electrophoresis (Invitrogen Life Technologies, Carlsbad, CA, USA). RNA integrity was evaluated using the Agilent 2200 Tape Station (Agilent Technologies, Palo Alto, CA, USA) and each sample had a RIN^e^ above 7.0. After removal of rRNA with the Epicentre Ribo-Zero rRNA Removal Kit (Illumina, Santiago, CA, USA), the purified RNAs were fragmented to approximately 200 bp. Subsequently, the fragmented RNA was amplified and transcribed into fluorescent cRNA according to the manufacturer’s protocol using the TruSeq^®^ RNA LT/HT Sample Prep Kit (Illumina, Santiago, CA, USA) The purified library products were evaluated using the Agilent 2200 TapeStation and Qubit^®^2.0(Life Technologies, Carlsbad, CA, USA). Each of the purified RNA samples showed an A260:A230 ratio above 2.0 and an A260:A280 ratio above 1.8, suggesting that the RNAs were sufficiently pure for RNA-seq.

### RNA-seq and data analysis

RNA was purified from brain tissue using TRIzol, amplified and transcribed into fluorescent cRNA, and the purified library products were diluted to 10 pM. RNA-seq was performed using an Illumina HiSeq 2500 (Illumina, Santiago, CA, USA), at Guangzhou RiboBio Co., Ltd. (Guangzhou, China). Paired-end reads were aligned to the mouse transcriptome with Tophat2 as previously described^50^. RNA-seq data were normalized for GC (guanine-cytosine) content with EDASeq software. The whole samples expression levels were presented as RPKM (expected number of Reads Per Kilobase of transcript sequence per Million base pairs sequenced), which is the recommended and most common method to estimate the level of gene expression^51^. The gene-level RPKM values were then normalized using the log10 values (RPKM + 1) for further analyses. Differential expression was determined with DEGseq software, and the *q-*value was used to denote the significance of the *P-*value (*q-*value < 0.05 is recommended)^52^. The data were deposited in Gene Expression Omnibus (GEO) with accession number GSE79416.

### Bioinformatics analysis

All differentially expressed mRNAs were selected for GO and KEGG pathway analyses to investigate the potential role of the lncRNAs**-**coexpressed with mRNAs. GO was performed with KOBAS2.0 software. GO provides label classification of gene function and gene product attributes (http://www.geneontology.org). GO analysis covers three domains: cellular component (CC), molecular function (MF) and biological process (BP)^53^. The false discovery rate (FDR) was used to denote the significance of the *P-*value (FDR < 0.05 is recommended). The differentially expressed mRNAs and the enrichment of different pathways were mapped using the KEGG pathways with KOBAS2.0 software (http://www.genome.jp/kegg)^54^,^55^. The significance of the KEGG pathways among differentially expressed genes was denoted by the FDR (FDR < 0.05 is recommended).

### Co-expression network of differentially expressed lncRNAs/mRNAs

To investigate the potential functions of differentially expressed lncRNAs and the interactions between mRNAs and lncRNAs, we constructed a lncRNA/mRNA transcripts co-expression network. We calculated the Pearson correlation coefficient (PCC), and the COR-value was used to calculate the correlation coefficient of the PCC between lncRNA and mRNA transcripts (not including lncRNA-lncRNA transcripts or mRNA-mRNA transcripts PCC). Considering the small sample (n < 8) and stochastic factors, the permutation test was performed. The Z score values were used to normalize the PCC, and then the *P-*value was calculated^56^ (|COR| > 0.95 and *p-*value < 0.05 is recommended). The co-expression network was illustrated using Cytoscape software^57^.

### Cell culture and treatments

The BV-2 mouse microglial cell line was cultured in Dulbecco’s modified Eagle’s medium (DMEM, Gibco, Grand Island, CA, USA) supplemented with 10% foetal bovine serum (FBS, Gibco, Grand Island, CA, USA) and 50 mg/mL of penicillin-streptomycin and maintained in ahumidified incubator at 37 °C, with 5% CO_2_. Seventy-two hours after the lentiviral vectors were transfected into BV-2 cells, LPS (Sigma, St. Louis, MO, USA) at a concentration of 1 μg/mL was added to cells of the LPS group. Then, the cells were cultured for 24~48 h. The HT22 mouse neuronal cell line and MA astrocyte cell line were cultured in DMEM-F12 (Gibco, Grand Island, CA, USA), and the MOPC oligodendrocyte precursor cell line was cultured in MM (Gibco, Grand Island, CA, USA), supplemented with 10% FBS (Gibco, Grand Island, CA, USA) and 50 mg/mL of penicillin-streptomycin. Cells were maintained in a humidified incubator at 37 °C, with 5% CO_2_.

### Lentiviral vector production and infection

Three different lentiviral vectors (termed 50305, 50306 and 50307; Genechem, Shanghai, China) targeting lncRNA fantom3_F730004F19 were transfected into BV-2 cells with the polybrene lentiviral vector transfection reagent (Roche, Mannheim, Germany). Non-transfected cells were used as the Blank control, negative control lentivirus was transfected as the negative control (NC), and the lncRNA fantom3_F730004F19 shRNA lentiviruses were transfected for the knock down (KD) groups. The sequences of the 3 lentiviral vectors were as follows:

Lentivirus-50305(KD1): TTCCTAAGGACTGGAAACATA

Lentivirus-50306(KD2): GAGGACAAGTCTGGAAGTCAA

Lentivirus-50307(KD3): TGACACAGGGCTACAGGGTAT

The efficacy of lentivirus transfection was evaluated by qRT-PCR.

### Validation of gene expression by qRT-PCR

To identify the veracity of sequencing, we randomly selected 5 lncRNA transcripts for validation. Total RNA was extracted from the control and SAH brain samples using TRIzol reagent (Invitrogen Life Technologies, Carlsbad, CA, USA) and then reverse transcribed to cDNA. Five randomly selected lncRNAs and their expression levels were further assessed by qRT-PCR using iQ^TM^ SYBR^®^ Green Supermix (Bio-Rad Laboratories, Inc. Hercules, CA, USA) with a CFX96 Touch™ Real-Time PCR instrument (Bio-Rad Laboratories, Inc. Hercules, CA, USA). To identify the silencing efficiency of the lentivirus and the influence of target genes, the total RNA was extracted from each group of BV-2 cells. All samples were normalized to the expression of *β*-actin, and the experiment was repeated 3 times. The detailed primer information is available as supplemental data (Supplementary Table S1).

### Fluorescence *in situ* hybridization experiments (FISH)

FISH staining was performed as previously described^58^. Briefly, BV-2 cells were washed twice with 1X PBS and then fixed with 4% paraformaldehyde for 10 min. After washed 3 times with 1X PBS, the cells were permeabilized with 0.5% Triton X-100 for 10 min. The cells were treated with pre-hybridization buffer for 30 min at 37 °C and then the appropriate amount of probe in a hybridization solution was applied overnight in a humidified chamber at 37 °C. Cells were then washed twice for 30 min at 42 °C with 0.1% Tween-20 in 4X SSC. DAPI was applied during the second wash. Cells were then rinsed twice with 1X PBS before imaging. Images were captured with afluorescence microscope (Eclipse Ti-S; Nikon, Tokyo, Japan).

### Western blotting analyses

Cells were washed twice with chilled PBS and lysed directly in wells by incubating with RIPA lysis buffer supplemented with a protease inhibitor (Roche, Basel, Switzerland) for 120 h post-transfection. The primary antibodies included mouse monoclonal antibody against CD14 (60253-1-Ig, Proteintech, Wuhan, China), mouse monoclonal antibody against TLR4 (ab 8376, Abcam, Cambridge, UK), and mouse monoclonal anti-beta-actin (#SC-47778, Santa Cruz, CA, USA).

### Immunofluorescence staining

Immunofluorescence staining was conducted as previously described^35^. Primary antibodies included mouse monoclonal antibody against CD14 (1:200, 60253-1-Ig, Proteintech, Wuhan, China), mouse monoclonal antibody against TLR4 (1:200, ab 8376, Abcam, Cambridge, UK). Secondary antibodies included a DyLight 488, goat anti-mouse IgG (A23210, Abbkin, California, USA). Nuclei were stained with 40′, 6-diamidino-2-phenylindole (C1006; Beyotime, Nanjing, China). Images were captured with afluorescence microscope (Eclipse Ti-S; Nikon, Tokyo, Japan). Image-pro plus (IPP) 6.0 software was used for immunofluorescence staining analysis.

### Enzyme-linked immunosorbent assay (ELISA)

The protein levels of IL-1β, IL-6 and TNF-α in the BV-2 supernatants were quantified by ELISA according to the manufacturer’s instructions (Boster, Wuhan, China). The relative protein content of IL-1β, IL-6 and TNF-α was shown as picogram per milligram of total protein. The protein content of each supernatants sample was detected with a BCA kit (Beyotime, Shanghai, China).

### Statistical analysis

All data are presented as the mean ± SEM unless otherwise stated. Comparisons between two groups were analysed using the Student’s t-test. *P* < 0.05 was considered to indicate a statistically significant difference.

## Additional Information

**How to cite this article**: Peng, J. *et al*. High-Throughput Sequencing and Co-Expression Network Analysis of lncRNAs and mRNAs in Early Brain Injury Following Experimental Subarachnoid Haemorrhage. *Sci. Rep.* **7**, 46577; doi: 10.1038/srep46577 (2017).

**Publisher's note:** Springer Nature remains neutral with regard to jurisdictional claims in published maps and institutional affiliations.

## Supplementary Material

Supplementary Information

Supplementary Table S1

Supplementary Table S2

Supplementary Table S3

Supplementary Table S4

Supplementary Table S5

## Figures and Tables

**Table 1:** Mapping of sequence reads in the control and SAH groups.

	Control	SAH
mapped statistics	Percentage	Percentage
effective Reads	110,198,118(100%)	110,540,492(100%)
Total mapped	106,601,985(96.74%)	107,043,049(96.84%)
Multiple mapped	7,834,333(7.11%)	8,447,917(7.64%)
Uniquely mapped	98,767,652(89.63%)	98,595,132(89.19%)
Read1 mapped	53,336,031(48.40%)	53,560,604(48.45%)
Read2 mapped	53,265,954(48.34%)	53,482,445(48.38%)
Reads map to ‘+’	53,511,203(48.56%)	53,734,014(48.61%)
Reads map to ‘−’	53,090,782(48.18%)	53,309,035(48.23%)
Reads mapped in proper pairs	52,517,680(47.66%)	52,720,928(47.69%)
exonic	78.73%	82.94%
intronic	16.27%	12.11%
intergenic	4.73%	4.66%
splicing	0.27%	0.29%

**Table 2:** Top 10 up-regulated and 10 down-regulated new lncRNAs.

New lncRNA ID	Position	Strand	Length	log2^(FoldChange)^	*p* value	*q* value
**TOP 10 upregulated**						
TCONS_00134940	chr2:152132482–152133792	+	1311	3.579309818	7.54E-10	4.25E-07
TCONS_00014609	chr1:164770824–164772107	+	1284	3.435888747	2.13E-13	2.79E-10
TCONS_00229655	chr8:13210965–13212785	−	611	3.407071645	6.35E-05	0.0037973
TCONS_00013698	chr1:164771315–164772344	+	1030	3.105719533	5.63E-10	3.29E-07
TCONS_00171085	chr4:123835876–123837032	+	1157	3.10367252	5.10E-07	0.0001139
TCONS_00155436	chr3:156242819–156372282	−	1389	2.863694799	1.01E-05	0.00106288
TCONS_00243215	chr9:109532586–109532943	+	358	2.854429136	0.0033574	0.04845512
TCONS_00155437	chr3:156242819–156411321	−	1454	2.753874179	2.67E-05	0.00212182
TCONS_00229452	chr8:122706260–122707523	+	1264	2.673461266	2.64E-06	0.00039418
TCONS_00141564	chr2:168116736–168117782	+	1047	2.546936401	2.03E-06	0.00032681
**TOP 10 downregulated**						
TCONS_00134739	chr2:124458566–124459471	+	906	−2.785739111	0.0022792	0.03815687
TCONS_00102546	chr17:49108855–49109859	+	1005	−2.845027227	3.41E-05	0.00249637
TCONS_00109537	chr18:45513359–45514133	+	775	−2.890310541	0.0010126	0.02281791
TCONS_00020217	chr10:9600630–9601340	+	711	−3.09197587	0.0015767	0.03014613
TCONS_00066295	chr14:76391379–76392214	+	836	−3.138016002	0.0010076	0.02275018
TCONS_00065182	chr14:66905035–66905822	+	788	−3.208470573	0.0014353	0.02846326
TCONS_00032471	chr11:96877961–96878982	+	1022	−3.289274477	1.57E-07	4.31E-05
TCONS_00212000	chr7:63986873–64011804	+	443	−3.519838355	0.0014797	0.02898882
TCONS_00227229	chr8:31932151–31932751	+	601	−3.684227245	0.0021418	0.03668885
TCONS_00209024	chr7:64017474–64019516	+	866	−4.152719101	0.0004011	0.01282738
