# Supplementary material for: High-Throughput Sequencing and Co-Expression Network Analysis of lncRNAs and mRNAs in Early Brain Injury Following Experimental Subarachnoid Haemorrhage
Source: Sci Rep. 2017 Apr 18;7:46577. doi: 10.1038/srep46577 (PMC5394545; doi:10.1038/srep46577)
Supplement: Supplementary Information [file srep46577-s1.pdf]

High-Throughput Sequencing and Co-Expression Network Analysis of lncRNAs and mRNAs in Early Brain Injury Following Experimental Subarachnoid Haemorrhage

Jianhua Peng[1], Yue Wu[2], Xiaocui Tian[3], Jinwei Pang[1], Li Kuai[4], Fang Cao[5], Xinghu Qin[1,6], Jianjun Zhong[2], Xinshen Li[1], Yong Li[1], Xiaochuan Sun[2], Ligang Chen[1] & Yong Jiang[1]

1 Department of Neurosurgery, the Affiliated Hospital of Southwest Medical University, Luzhou, China.
2 Department of Neurosurgery, the First Affiliated Hospital of Chongqing Medical University, Chongqing, China.
3 Chongqing Key Laboratory of Biochemistry and Molecular Pharmacology, College of Pharmacy, Chongqing, China.
4 Department of Ophthalmology, the Affiliated Hospital of Southwest Medical University, Luzhou, Sichuan, China.
5 Department of Neurovascular Disease, the Affiliated Hospital of Zunyi Medical College, Zunyi, China.
6 Department of Neurosurgery, People's Hospital of Deyang City, Deyang, China. Correspondence and requests for materials should be addressed to Y.J. (E-mail: jiangyong@swmu.edu.cn)

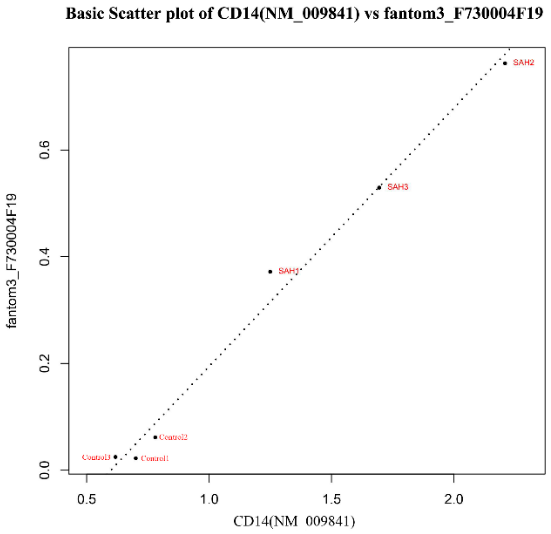

utlier_sample	rstudent	unadjusted p-value	Bonferonni p
SAH1	2.958601	0.06	0.35767

Figure S1. Plots of CD14 and fantom3_F730004F19. (A) Plot for the expression of CD14 versus the expression of fantom3_F370004F19. The plot suggested that SAH1 sample might be an outliers. (B) The Outlier Test for SAH1 sample. The results suggested that both the unadjusted p-value and bonferonni p-value (corrected p-value) were greater than 0.05, suggested that the correlation coefficient was credible (The larger of p value, the lower probability of outliers that the sample is).

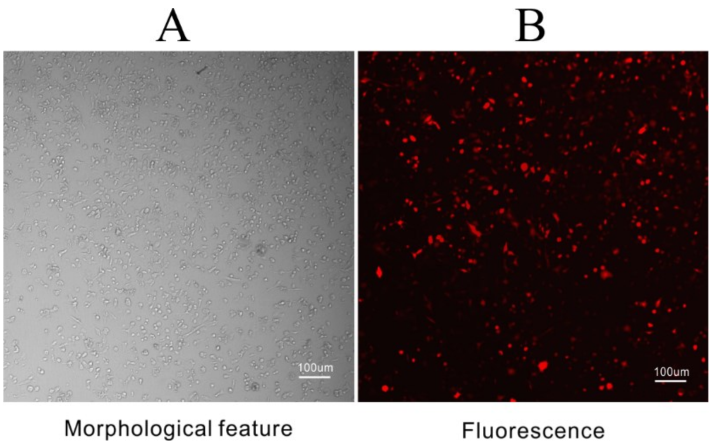

Figure S2. Lentivirus transfection of fantom3_F730004F19. (A) Morphological feature of BV-2 microglia cells. (B) Cells were well transfected with Cherry Fusion Tag Lentiviral Vector (red).

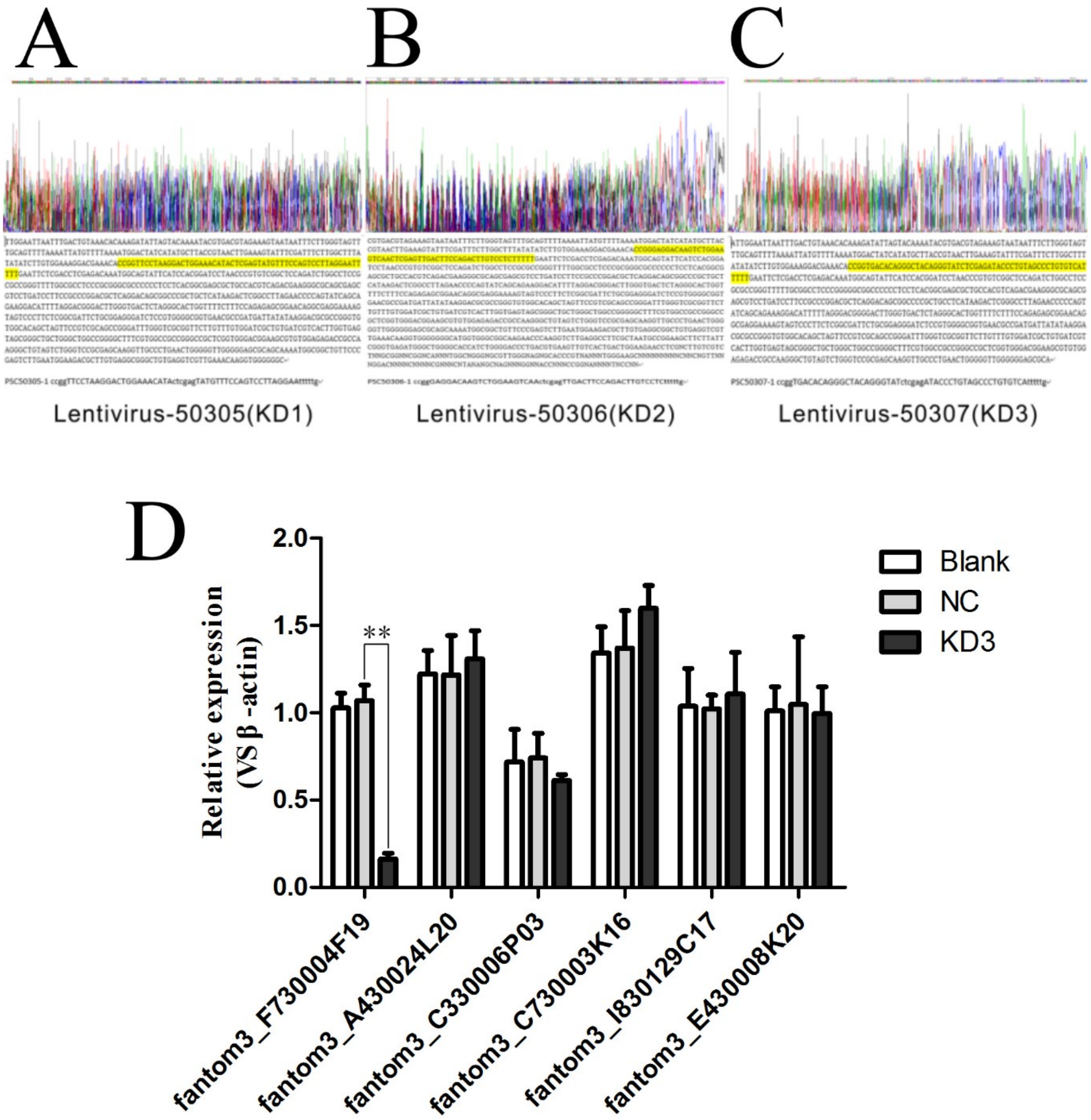

Figure S3. Lentivirus knockdown specific of fantom3_F730004F19. Sequencing technique were performed to compare the sequencing results with gene sequence. The results indicated that all the lentivirus sequence were well matched with lncRNA fantom3_F730004F19. A: Lentivirus-50305(KD1); B: Lentivirus-50306(KD2); C: Lentivirus-50307(KD3). qRT-PCR results indicated that lentivirus-50307(KD3) couldn't knockdown other lncRNAs. KD3 present a specific lentiviral-mediated knockdown of this particular fantom3_F730004F19 (D). ** p<0.01.

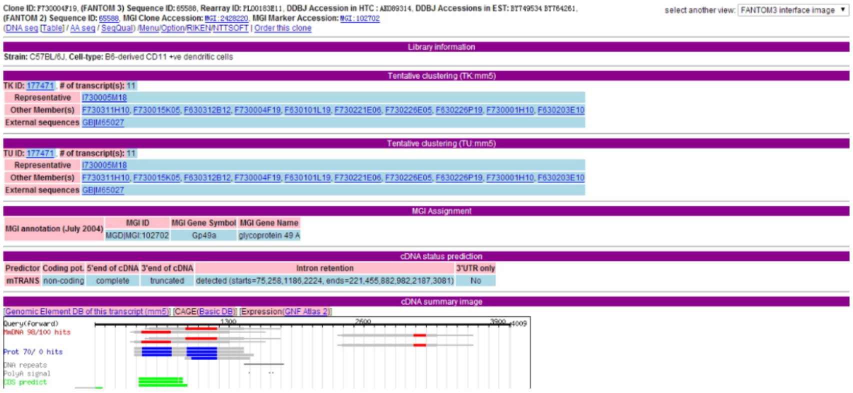

Figure S4. LncRNA fantom-f730004f19 information. The detailed information of lncRNA fantom-f730004f19 can be searched in fantom database (http://fantom.gsc.riken.jp).

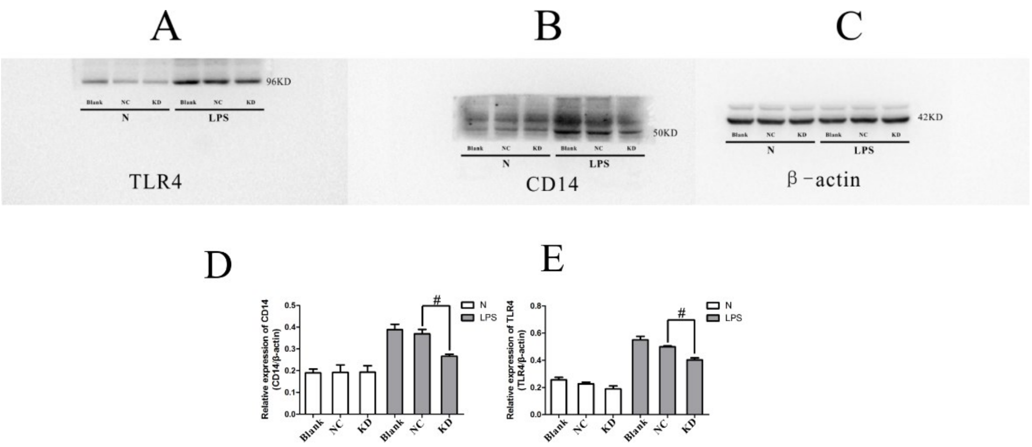

Figure S5. Full-length blots/gels of western blot analysis. The full-length and original contrast blots of TLR4 (A, D), CD14 (B, E) and β-actin (C) of western blot analysis. N: normal condition group; LPS: lipopolysaccharide treated; Blank: non-transfected cells; NC: negative control lentivirus transfected cells; KD: the lncRNA fantom3_F730004F19 lentivirus transfected cells.
